# Anti-Inflammatory Effect of Gamisoyo-San in an Animal Model of Amyotrophic Lateral Sclerosis

**DOI:** 10.1155/2018/8580152

**Published:** 2018-06-21

**Authors:** Sunjung Park, Eun Jin Yang

**Affiliations:** Department of Clinical Research, Korea Institute of Oriental Medicine, 1672 Yuseong-daero, Yuseong-gu, Daejeon 305-811, Republic of Korea

## Abstract

Inflammation is considered a critical factor in the pathogenesis of amyotrophic lateral sclerosis (ALS). We aimed to evaluate the effect of the herbal formula Gamisoyo-San (GSS) on the muscles of hSOD1^G93A^ transgenic mice, a mouse model of ALS, by examining the tissue expression of inflammation- and oxidative stress-related proteins. The mice were randomly divided into three groups: nontransgenic mice (non-Tg, n = 4), hSOD1^G93A^ transgenic mice (Tg, n = 4), and GSS-treated hSOD1^G93A^ transgenic mice (Tg+GSS, n = 4). Eight-week-old female hSOD1^G93A^ transgenic mice were fed GSS (1 mg/g body weight) for 6 weeks. Gastrocnemius (GA) tissues were analyzed for inflammatory proteins [CD11b and toll-like receptor 4 (TLR4)] and oxidative stress-related proteins [heme oxygenase 1 (HO1) and ferritin] by western blot analysis. Administration of GSS significantly reduced the level of inflammation- and oxidative stress-related proteins in hSOD1^G93A^ transgenic mice. GSS ameliorated inflammation by downregulating TLR4 and CD11b expression and regulated iron homeostasis in the GA muscle of hSOD1^G93A^ mice. GSS could help reduce inflammation by regulating immune reactions in patients with ALS. To the best of our knowledge, this is the first study to demonstrate the effect of GSS on muscle inflammation in an ALS animal model.

## 1. Introduction

Amyotrophic lateral sclerosis (ALS) is a neurological disorder characterized by the degeneration of cranial, brainstem, and spinal motor neurons, leading to death within 3–5 years after disease onset in humans [1]. There have been numerous studies on the pathogenesis and cellular mechanisms of ALS, which have implicated the involvement of inflammation, oxidative stress, axonal transport impairment, mitochondrial defects, and protein aggregation [2].

In approximately 10% of ALS patients, the disease is inherited and caused by gene mutations, including mutations in superoxide dismutase 1 (SOD1), TAR DNA-binding protein 43 (TDP43), C9orf72, Optineurin (OPTN), P62 (Sequestosome 1, SQSTM1), Ubiquilin 2 (UBQLN2), TANK-binding kinase 1 (TBK1), and fused in sarcoma (FUS). SOD1 mutations are highly prevalent and observed in 20% of familial ALS and 2–7% of sporadic ALS cases [3]. Mouse models of ALS carrying gene mutations have been developed, and SOD1 mutant mice recapitulate several core clinical as well as neuropathological features of ALS [4]. Mutant SOD1 transgenic models carry a G93A mutation in their human SOD1 transgene [5]. SOD1 mutant mice have motor ability defects and show progressive hind limb tremors and weakness, locomotor deficits, and paralysis, eventually resulting in death. Motor neuron degeneration interrupts neuron-to-muscle and muscle-to-neuron signaling, leading to severe muscle wasting and atrophy [6]. Gabriella et al. reported that the accumulation of reactive oxygen species (ROS) in the muscles of SOD1^G93A^ transgenic mice inhibits phosphoinositide 3-kinase (PI3K)/AKT signaling, resulting in the activation of Forkhead box O (FOXO) proteins, which in turn promotes muscle atrophy [7]. This suggests that inhibition of protein synthesis and increased protein catabolism via the PI3K/AKT pathway results in accelerated muscle atrophy in the early stages of ALS in SOD1^G93A^ transgenic mice [7]. Abnormal increases in autophagy-related proteins lead to autophagy dysfunction, impairment of lysosomal fusion, and enhanced inflammation in muscles [8]. The expression of several autophagy factors, including LC3 and P62, is increased in the skeletal muscle of SOD1^G93A^ transgenic mice [9, 10]. This suggests that autophagy dysfunction results in muscle inflammation in these mice.

The use of complementary and alternative medicine (CAM), especially in patients with chronic and incurable diseases, such as Alzheimer's disease (AD) [11] and Parkinson's disease (PD) [12], is increasing in all countries. Acupuncture, a type of CAM, was shown to be an effective therapy for AD and PD in both animal models and clinical trials [11, 12]. The drug riluzole has been approved for the treatment of patients with ALS in many countries. However, it reduces the lifespan of ALS patients by approximately 2–3 months [13]. Because of the side effects and high costs of riluzole, the majority of ALS patients consider CAM, including acupuncture, energy healing, and nutritional supplements [14]. Besides prolonging life, patients seek CAM to improve quality of life and attenuate their symptoms. Gamisoyo-San (GSS), a well-known herbal formula in CAM, is known to be beneficial in improving women's health, and it has been widely used by the Chinese and Koreans for thousands of years [15, 16]. GSS comprises* Bupleurum chinense* (1 g), Radix Angelicae sinensis (1 g), Paeoniae Radix alba (1 g), Rhizoma* Atractylodis macrocephalae* (1 g), Sclerotium Poriae cocos (1 g), Rhizoma Zingiberis officinalis recens (0.33 g),* Paeonia suffruticosa* Andrews (0.67 g),* Gardenia jasminoides* (0.67 g), Mentha haplocalyx (0.33 g), and* Glycyrrhiza uralensis* (0.67 g) [17]. GSS is often used to treat symptoms such as anxiety, anorexia, night sweating, headache, dry eyes, hot flushes, palpitations, and irregular menstruation [16, 18–20]. GSS is a very useful herbal formula for menopausal women, and it alleviates symptoms in women being treated for breast cancer [21]. A recent study reported that GSS inhibits inducible nitric oxide synthase (iNOS), cyclooxygenase 2 (COX2), and tumor necrosis factor-*α* (TNF-*α*) and exerts anti-inflammatory effects on macrophages [22]. In addition, some studies reported that GSS regulated stress-related neuropsychological disorders, such as depression and anxiety [15, 16, 19, 23]. However, the effects of GSS in the treatment of neuropsychological disorders or neurodegenerative diseases are not fully understood. In our previous studies, we demonstrated that herbal medicine, such as bee venom and* Scolopendra subspinipes mutilans, *attenuated neuroinflammation in symptomatic ALS mouse model [24–28]. Therefore, in the present study, we aimed to evaluate whether GSS treatment reduces muscle inflammation in female SOD1^G93A^ transgenic mice. We have already investigated the effects of GSS on neuroinflammation in the spinal cord of symptomatic ALS mouse model (unpublished data).

## 2. Materials and Methods

### 2.1. Materials

The antibodies used in this study were obtained as follows: rabbit anti-toll-like receptor 4 (TLR4), anti-BAX, and goat anti-Actin were purchased from Santa Cruz Biotechnology, Inc. (Dallas, TX, USA); rabbit anti-heme oxygenase-1 (HO1) and anti-ferritin were purchased from Abcam (Abcam, MA, USA); and all secondary antibodies were purchased from Santa Cruz Biotechnology.

### 2.2. Animals and Genotyping

The human SOD1^G93A^ transgenic mouse strain is considered a well-established animal model for human ALS. Hemizygous transgenic B6SJL mice (hSOD1^G93A^) were originally obtained from the Jackson Laboratory (Bar Harbor, ME, USA) and in-house breeding was carried out in our animal facility. Transgenic mice were identified by polymerase chain reaction (PCR) as described previously [29]. The mice were allowed access to water and food* ad libitum* and were maintained at a constant temperature (21 ± 2°C) and humidity (50 ± 10%) with a 12-h light/dark cycle (light on 07:00–19:00). All mice were handled in accordance with the animal care guidelines of the Korea Institute of Oriental Medicine (#15-036) and National Institutes of Health (NIH) for use of laboratory animals.

### 2.3. GSS Administration

GSS was purchased from Hankookshinyak (Chungnam, Korea) and dissolved in autoclaved distilled water. Eight-week-old female mice were randomly divided into three groups: nontransgenic mice (non-Tg, n = 4), hSOD1^G93A^ transgenic mice (Tg, n = 4), and GSS-administered SOD1^G93A^ transgenic mice (GSS-treated Tg, n = 4). GSS was administered once daily for 6 weeks, at a daily oral dose of 1 mg/g body weight.

### 2.4. Western Blot Analysis

After administration of GSS for 6 weeks, 14-week-old female mice were anesthetized with an intraperitoneal injection of pentobarbital (2.5 mg/g) and perfused with phosphate-buffered saline (PBS). The gastrocnemius (GA) muscles of the hind limb were dissected and homogenized in RIPA buffer (50 mM Tris-HCl [pH 7.4], 1% NP-40, 0.1% sodium dodecyl sulfate [SDS], and 150 mM NaCl) containing a protease inhibitor cocktail (Thermo Fisher Scientific, MA, USA). After homogenization, the protein concentration was determined using bicinchoninic acid (BCA) assay (Thermo Fisher, MA, USA). Western blotting was performed as previously described [27]. In brief, total protein (20 *μ*g) was denatured in sodium dodecyl sulfate (SDS) sample buffer, separated by SDS-polyacrylamide gel electrophoresis (PAGE), and transferred to a polyvinylidene fluoride (PVDF) membrane (Bio-Rad, CA, USA). For detection of target proteins, the membranes were blocked using 5% nonfat milk in TBS (50 mM Tris-Cl [pH 7.6], 150 mM NaCl) and then incubated overnight separately with the following primary antibodies: anti-Actin, anti-BAX, anti-TLR4, anti-CD11b, anti-ferritin, or anti-HO1. The blots were washed, probed with peroxidase-conjugated secondary antibodies, washed, and then visualized using enhanced chemiluminescence reagents (Amersham Pharmacia, NJ, USA). The protein bands were detected using a Fusion SL4 imaging system (Fusion, Eberhardzell, Germany).

### 2.5. Statistical Analysis

All data were analyzed using GraphPad Prism 5.0 software (GraphPad Software, CA, USA) and are presented as means ± standard error of the mean (SEM), where indicated. An ANOVA test was used to compare the differences among non-Tg, Tg, and GSS-treated Tg groups. Statistical significance was set at p<0.05.

## 3. Results

### 3.1. GSS Regulates the Expression of Inflammatory Proteins in the GA Muscle of hSOD1^G93A^ Mice

**Figure 1:**
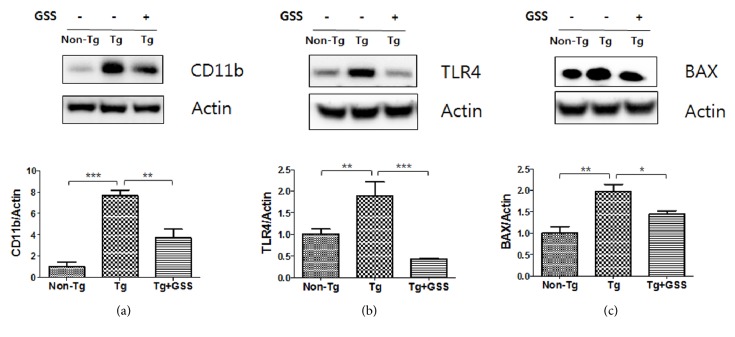
**Effect of Gamisoyo-San (GSS) on muscle inflammation in** **S** **O** **D**1^**G**93**A**^** mice**. GSS reduces inflammatory protein levels in the gastrocnemius (GA) muscle of SOD1^G93A^ mice. Equal amounts of muscle lysates from mice in non-Tg, Tg, and GSS-treated Tg groups (n= 4/group) were subjected to western blot analysis for (a) CD11b, (b) TLR4, or (c) BAX. Data represent the means ± SEM. Statistical significance was calculated by ANOVA, *∗*p<0.05, *∗∗*p<0.01, *∗∗∗*p<0.001.

To determine whether GSS administration regulates inflammatory protein expression, we evaluated the effects of GSS on CD11b, TLR4, and BAX levels in the GA muscles of hSOD1^G93A^ mice using western blot analysis. As shown in Figure 1(a), we found that the expression of CD11b (7.6-fold), a marker of inflammatory protein, in the muscle of Tg mice was upregulated compared with that in non-Tg mice. GSS treatment reduced the levels of CD11b by 2.0-fold in the GA muscle of hSOD1^G93A^ mice. In addition, we found that the expression of TLR4 (1.9-fold) was upregulated in Tg mice compared to that in non-Tg mice (Figure 1(b)). The expression of TLR4 (4.0-fold) protein in GSS-treated Tg mice was significantly lower than that in untreated Tg mice. As shown in Figure 1(c), we confirmed that BAX levels were increased by 2.0-fold in the GA muscle of Tg mice compared with the levels in non-Tg mice. GSS treatment reduced BAX protein expression by 1.3-fold in the GA muscle of Tg mice. These results suggest that GSS treatment reduced inflammation in the muscles of ALS mouse model.

### 3.2. GSS Alleviates the Expression of Oxidative Stress-Related Proteins in the GA Muscle of hSOD1^G93A^ Mice

**Figure 2:**
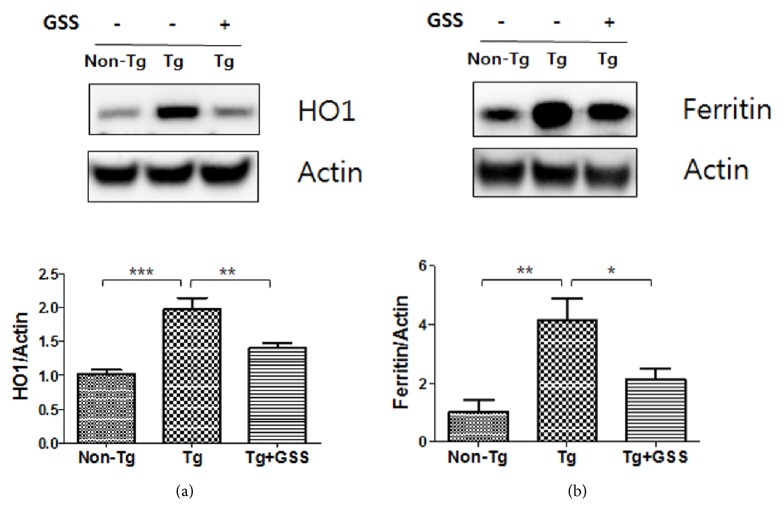
**Effect of Gamisoyo-San (GSS) on oxidative stress in the muscle of** **S** **O** **D**1^**G**93**A**^** mice**. GSS reduces oxidative stress-related proteins in the gastrocnemius (GA) muscle of SOD1^G93A^ mice. Representative images show immunoblots for (a) heme oxygenase 1 (HO1) or (b) ferritin in lysates from the GA muscle of mice in non-Tg, Tg, and GSS-treated Tg groups (n= 4/group). Data represent the means ± SEM. Statistical significance was calculated by ANOVA, *∗*p<0.05, *∗∗*p<0.01, *∗∗∗*p<0.001.

Oxidative stress results in the production of free heme. Free heme can be involved in the generation of ROS, which further increases oxidative stress. As shown in Figure 2(a), we found increased HO-1 expression (2.0-fold) in the GA muscle of untreated Tg mice compared with non-Tg mice. GSS-treated Tg mice had 1.4-fold lower HO-1 levels in GA muscle than untreated Tg control mice did. In previous studies, increase in the activity of iron-responsive element-binding proteins in response to oxidative stress was observed [30, 31]. Ferritin is a useful marker that reflects the importance of oxidative stress. As shown in Figure 2(b), we found increased ferritin expression (4.0-fold) in the GA muscle of Tg control mice compared with non-Tg mice. GSS-treated Tg mice had 1.9-fold lower ferritin levels in GA muscle than Tg control mice did. These findings suggest that GSS exerts antioxidative effects in the muscles of SOD1^G93A^ mice.

## 4. Discussion

In this study, we investigated the effect of GSS on muscle inflammation in hSOD1^G93A^ mice. We found that GSS reduced inflammatory protein expression and oxidative stress in the muscles of these transgenic mice. To the best of our knowledge, this study is the first to show that GSS can prevent inflammation-induced muscle atrophy in the muscles of mice with neurodegenerative disease.

The pathological mechanisms of ALS have been demonstrated in the central nervous system (CNS), including the spinal cord and brain [32]. Since muscle atrophy is the endpoint and muscle weakness ultimately leads to death in ALS patients, many studies have focused on alleviating atrophy and weakness in ALS animal models. Léger et al. found that an increase in expression of the E3 ubiquitin ligase atrogin 1 contributed to skeletal muscle atrophy [33]. Dobrowolny et al. demonstrated that muscle atrophy was induced by FOXO3 induction and upregulation of autophagic pathway proteins, including microtubule-associated protein 1A/1B-light chain 3 (LC3), BCL2/adenovirus E1B 19 kDa protein-interacting protein 3 (Bnip3), and cathepsin L, in ALS animal models [7]. Notably, the overexpression of insulin-like growth factor (IGF) in skeletal muscles delayed disease progression and increased survival in an ALS model [34, 35]. In addition, some studies have shown that the delivery of stem cell-based growth/trophic factors to the skeletal muscle increased lifespan and disease progression in ALS models [36, 37]. From these results, we suggest that therapeutic approaches to improve muscle function would effectively delay disease progression and increase survival in ALS animal models and possibly ALS patients.

Inflammation is one of the major pathological factors in motor neuron death, and it leads to motor neuron degeneration in the CNS [38]. In ALS, inflammation increases in the skeletal muscle and neuromuscular junction (NMJ) with strong activated glial responses [39].

In this study, we examined the effect of GSS on inflammation in the skeletal muscle of transgenic mice carrying the mutant hSOD1^G93A^ gene. The levels of inflammatory markers CD11b and TLR4 significantly increased in the limb muscle of ALS mice. This increase in macrophage markers is similar to what has been previously observed in the spinal cord of hSOD1^G93A^ mice [28]. However, GSS reduced the levels of inflammatory markers, including TLR4, in the GA muscle of hSOD1^G93A^ mice. TLRs play a role in the innate immune system, which mediates the expression of proinflammatory cytokines, such as TNF-*α*, in several cell types and tissues [40]. In addition, TLRs induce inflammatory responses through NF-kB signaling in skeletal muscle [41]. Verzola et al. demonstrated the upregulation of TLR4 signaling, including TNF-*α*- and NF-*κ*B-regulated genes that promoted muscle inflammation [42].

Oxidative stress is associated with inflammation and can damage muscle fibers [43]. For example, superoxide can activate redox-sensitive transcription factors, such as NF-*κ*B, that are involved in the production of inflammatory mediators such as interleukin-1*β* (IL-1*β*), IL-6, TNF-*α*, and COX2 [44]. Therefore, antioxidants can modulate inflammation in skeletal muscle. Some reports have indicated that vitamin C, flavonoids, and anthocyanins reduce oxidative stress and inflammation [45–47]. In this study, we showed that GSS inhibited the expression of oxidative stress-related proteins (HO1 and ferritin) in the muscle of hSOD1^G93A^ mice. Our study also suggests that GSS acts as an antioxidant in muscle tissues.

GSS in Korea, Jia Wei Xiao Yao San (JWXYS) in Chinese herbal medicine (CHM), and kami-shoyo-san in Japanese traditional medicine are widely used to treat stress-related neuropsychological disorders, such as depression and anxiety, and hot flashes in peri- and postmenopausal women [19, 48, 49]. Although the mechanism underlying the effects of GSS in neuropsychological disorders or hot flashes is not fully understood, Yasui et al. suggested that the decrease in IL-6 levels by kami-shoyo-san in women with hot flashes may occur via regulation of the hypothalamic-pituitary-adrenocortical axis and feedback inhibition [19]. Lin et al. also reported that JWXYS treatment regulates smooth cell contractility in diabetic patients [50].

Although this is the first study to demonstrate the effects of GSS on muscle inflammation in an ALS animal model, further study on its effects on NMJ and spinal cord in hSOD1^G93A^ mice is warranted because ALS is a neuromuscular disorder. In addition, the active constituents of GSS that inhibit inflammation and oxidative stress in the muscles of hSOD1^G93A^ mice need to be identified in future research.

## 5. Conclusions

In summary, we demonstrated that GSS significantly reduced the levels of inflammation- and oxidative stress-related proteins in hSOD1^G93A^ transgenic mice. GSS effectively ameliorated the expression levels of TLR4, CD11b, and BAX in the GA muscle of SOD1^G93A^ mice. In addition, GSS regulated iron homeostasis via HO-1 and ferritin in the GA muscle of hSOD1^G93A^ mice. These findings suggest that GSS could help reduce inflammation and oxidative stress by regulating immune systems in ALS patients.

## Data Availability

Data supporting the conclusions of this research are contained in the article.
